# Body mass index and health status in diabetic and non-diabetic individuals

**DOI:** 10.1038/nutd.2015.2

**Published:** 2015-04-27

**Authors:** A Jerant, K D Bertakis, P Franks

**Affiliations:** 1 UC Davis School of Medicine, Department of Family and Community Medicine and Center for Healthcare Policy and Research, Sacramento, CA, USA

## Abstract

**Background/Objectives:**

There is controversy regarding the existence of a body mass index (BMI) mortality paradox in diabetes, whereby the optimal BMI category is higher than it is in non-diabetic persons. To explore possible pathways to a mortality paradox, we examined the relationship of BMI with physical and mental health status in diabetic and non-diabetic persons.

**Subjects/Methods:**

We examined adjusted SF-12 Physical and Mental Component Summary (PCS-12 and MCS-12) scores by BMI (kg m^−2^) category (underweight, <20; normal weight, 20 to <25; overweight, 25 to <30; obese, 30 to <35; severely obese ⩾35) in adult diabetic and non-diabetic respondents to the 2000–2011 United States national Medical Expenditure Panel Surveys (*N*=119 161). Adjustors were age, sex, race/ethnicity, income, health insurance, education, smoking, comorbidity, urbanicity, geographic region and survey year.

**Results:**

In non-diabetic persons the adjusted mean PCS-12 score was highest (that is, most optimal) in the normal-weight category, whereas for diabetic persons the optimal adjusted mean PCS-12 score was in the overweight category (adjusted difference between non-diabetic and diabetic persons in the difference in PCS-12 means for overweight versus normal-weight category=0.8 points, 95% confidence interval; CI 0.1, 1.6; *P*=0.03). This paradoxical pattern was not evident for the MCS-12, and the adjusted difference between non-diabetic and diabetic persons in the difference in MCS-12 means for overweight versus obese persons was not significant (−0.3 points, 95% CI −0.9, 0.4; *P*=0.43). The findings were not significantly moderated by smoking status, cancer diagnosis or time period.

**Conclusions:**

The optimal BMI category for physical health status (but not mental health status) was higher among diabetic than non-diabetic persons. The findings are consistent with a BMI physical health status paradox in diabetes and, in turn, a mortality paradox.

## Introduction

Most studies examining the relationship of body mass index (BMI) with mortality in diabetic persons suggest a paradox: the BMI category with lowest associated mortality risk (overweight or obese) is higher than it is in non-diabetic persons (normal weight).^1, 2, 3, 4, 5, 6, 7^ Though apparently counterintuitive, genetic and physiological differences between leaner and heavier diabetic individuals could account for a BMI mortality paradox in diabetes,^1, 4, 8, 9, 10, 11^ particularly among those with Type 2 diabetes,^12^ which accounts for most cases in adults. However, in one high-profile study limited to persons with incident diabetes, normal weight was associated with the lowest mortality risk,^13^ re-igniting the long-standing controversy regarding the existence and potential health implications of a BMI mortality paradox in diabetes.^14, 15, 16^

Unstudied is whether a BMI health status paradox exists in diabetes, analogous to the BMI mortality paradox. Addressing this question is important in gauging the current net health impact of overweight and obesity in diabetes, and also has implications for the BMI mortality paradox debate, as health status influences mortality risk.^17, 18, 19, 20, 21, 22^ Heavier diabetic persons could have better health status than their leaner counterparts, owing to the same factors invoked in explaining the BMI mortality paradox,^1, 8, 9, 10^ or to uncharacterized factors. Alternatively, health status could be worse among heavier diabetic persons,^23, 24, 25^ owing to excess weight or its sequelae (for example, other metabolic conditions, osteoarthritis).^26, 27^

National studies comparing the health status of diabetic and non-diabetic persons across BMI categories are lacking. Prior studies of national samples show that health status tends to be lower among diabetic persons than among non-diabetic persons.^24, 28, 29^ However, the relationship of BMI with health status in diabetes is less clear, paralleling uncertainty regarding the relationship of BMI with mortality. Some studies find health status to be highest among normal-weight persons (including those with diabetes), whereas others find little influence of BMI on health status.^24, 28, 30, 31, 32, 33, 34, 35^ Reconciling these disparate findings is challenging owing to differences among the studies. Key differences include the type of health status examined (physical, mental, or overall), the degree of comorbidity adjustment, and the recency of the data employed, a particularly important issue given secular trends in BMI, diabetes and health status.^36, 37, 38, 39, 40, 41, 42^ As others have emphasized, the study of BMI paradoxes hinges on making comparisons between groups of individuals with and without a chronic health condition of interest.^43^ Thus, exploration of a possible BMI health status paradox in diabetes requires comparing physical and mental health status by BMI category in concurrent samples of diabetic and non-diabetic persons. Given that health status influences mortality risk,^17, 19, 21, 22^ including in diabetes,^18, 20^ the findings of such comparisons could suggest possible pathways to a BMI mortality paradox, informing the ongoing debate regarding its existence.

Using national data from the 2000–2011 United States national Medical Expenditure Panel Surveys (MEPS), we examined the relationship of BMI with physical and mental health status in diabetic and non-diabetic persons. The main analyses were adjusted for socio-demographic characteristics, chronic health conditions other than diabetes, smoking status and survey year (to account for secular trends). In additional analyses, we also explored whether smoking status, cancer diagnosis or time period moderated the relationship between BMI and diabetes and health status.

## Subjects and methods

The MEPS is an annual national survey of health-care use and costs in the civilian, non-institutionalized population in the United States, employing an overlapping panel design.^44^ The analytic sample for the current study included persons aged 18–90 years old at entry. The study was exempted by the University of California Davis Institutional Review Board.

In the MEPS, the Household Component includes information on respondent socio-demographics and health insurance, and a self-administered questionnaire includes items on smoking and health conditions. The full-year response rate varied from 70.5 to 59.4% for the 2000–2011 panels.^44^

### Measures

BMI in kg m^−2^ was constructed from self-reported height and weight. BMI categories employed in analyses were: <20 (underweight); 20–<25 (normal weight); 25–<30 (overweight); 30–<35 (obese); and ⩾35 (severely obese). These categories correspond to those widely employed by clinicians, except for the underweight and normal-weight categories, typically defined in clinical practice and most research prior to 2000 as <18.5 and 18.5–<25.^45^ A BMI of <20 was employed to distinguish underweight in the current analyses, as prior work indicates health status worsens sharply below that cut point, likely owing to the effects of concurrent illnesses.^46^ Classifying individuals with a BMI of 18.5–<20 as normal weight would artificially increase the risk of poor health status associated with normal weight and decrease the risk of poor health status associated with overweight and obesity.

Physical and mental health status were measured with the SF-12 Physical Component Summary (PCS-12) and Mental Component Summary (MCS-12) scales, respectively.^47^ Standardized scoring algorithms are employed to derive both scales, which range from 0 to 100, with higher scores indicating better self-rated health. Both summary scales were designed so that a representative sample of the US population would have a mean score of 50 with a standard deviation of 10.^47^

#### Health conditions and socio-demographics

Diabetes was self-reported (present or not), as were eight other chronic conditions: cancer, hypertension, coronary heart disease, myocardial infarction, cerebrovascular disease, asthma, emphysema and arthritis. Agreement between MEPS respondent-reported and clinician-reported health conditions is high.^48^ Self-reported smoking status was dichotomized as current smoker or not. Socio-demographic variables examined were: age in years; sex; race/ethnicity category (Hispanic (any race), non-Hispanic White, non-Hispanic Black or non-Hispanic other race); household income level as a percentage of Federal Poverty Level (<100%, 100–124%, 125–199%, 200–399%, or ⩾400%); health insurance status (uninsured, privately insured, or publicly insured); education level (less than high school (0–8 years of formal schooling), some high school (9–11years), high school graduate (12years), some college (13–15 years), or college graduate (⩾16 years)); US Census region (West, Midwest, Northeast, South); and urbanicity (living in a Metropolitan Statistical Area or not).

### Data analysis

Data were analyzed using Stata 13.1 (Stata Corporation, College Station, TX, USA), adjusting for the complex survey design of MEPS. Data were analyzed using longitudinal strata and primary sampling unit identifiers and survey weights, to derive estimates representative of the US civilian, non-institutionalized adult population. The primary analyses examined associations of BMI category (the key independent variable) with the PCS-12 and MCS-12 scales (the dependent variables), in linear regression models that included diabetes status (present versus absent) as well as a diabetes(present) × BMI category interaction term. All models adjusted for socio-demographic characteristics (age, age squared, sex, race/ethnicity (reference category=non-Hispanic White), household income as a percentage of Federal Poverty Level (reference⩽100%), health insurance status (reference=private insurance), education (reference=less than high school), Census region (reference=Northeast) and urban residence); chronic health conditions other than diabetes (from a count of eight conditions); smoking status; and MEPS survey year, included as a categorical variable (reference=2000). To explore whether key potential confounders (smoking, cancer, or study time period (2000–2005 versus 2006–2011)) moderated the relationships between BMI and diabetes and health status, we conducted additional analyses including three-way interaction terms: key confounder × diabetes status × BMI category.

To facilitate interpretation of the net adjusted associations between BMI category and health status, the findings of all models are presented as adjusted predictive marginal effects, which estimate of the amount of change in health status score produced per change in BMI category.^49^ We examine the difference between the marginal health status score (physical or mental) for the BMI category associated with the lowest score in diabetic persons with that found among normal-weight diabetic persons. We contrast that difference with the difference observed between the same BMI categories among non-diabetic persons. We present the resulting 'difference in difference' in the Results text.

## Results

There were 138 944 adults aged 18–90 entering the MEPS cohorts between 2000 and 2011; 119 161 (87.6%, population weighted) had complete data and were included in the current analyses. Table 1 summarizes the characteristics of the analytic sample by diabetes status. Compared with participants who did not report diabetes, those reporting diabetes were older and more likely to be Hispanic (any race) or non-Hispanic Black, have low household income and education, reside in the South and in non-urban areas, have more comorbid chronic health conditions, be non-smoking and be obese or severely obese. Participants reporting diabetes also had lower mean PCS-12 and MCS-12 scores than those not reporting diabetes.

Table 2 presents the unadjusted mean PCS-12 and MCS-12 scores of the analytic sample by BMI category and diabetes status. For both measures, across the range of study BMI categories, scores were lower among respondents with versus without diabetes. Among diabetic persons, for both measures the unadjusted mean scores were highest among those in the overweight category. By contrast, for non-diabetic persons, while unadjusted MCS-12 scores were again highest for those in the overweight category, unadjusted PCS-12 scores were highest in the normal-weight category.

**Figure 1:**
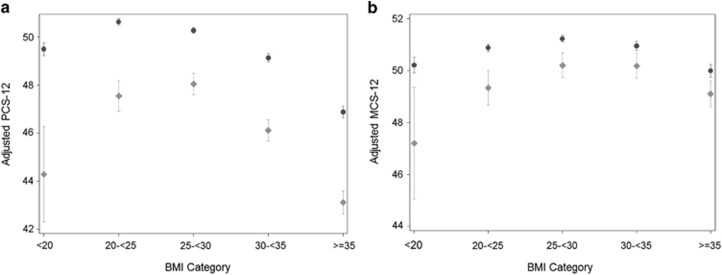
Adjusted physical and mental health status by body mass index category among diabetic versus non-diabetic persons (*N*=119 161). Legend: Panel **a**: physical health status (PCS-12). Panel **b**: mental health status (MCS-12). Dark circles: point estimates for non-diabetic persons. Light diamonds: point estimates for diabetic persons. Bars around point estimates indicate 95% confidence intervals. Analyses adjusted for age, age squared, gender, race/ethnicity, family income, education, insurance coverage, number of chronic health conditions, smoking status, rurality, region, and panel year. Abbreviations: MCS-12, SF-12 Mental Component Summary score; PCS-12, SF-12 Physical Component Summary score.

Table 3 and the Figure 1 show the adjusted PCS-12 and MCS-12 scores by BMI category and diabetes status. Across all study BMI categories, scores were lower for diabetic versus non-diabetic persons, with the most marked decrements for underweight diabetic persons. Among non-diabetic persons, for both health status measures, scores were lowest in the severely obese category. Among diabetic persons, the same pattern was observed for the PCS-12 (Table 3 and Figure 1, panel a), but for the MCS-12, scores were lowest in the underweight category (Table 3 and Figure 1, panel b).

For the PCS-12 (Table 3 and Figure 1, panel a), among non-diabetic persons scores were highest in the normal-weight group and significantly higher in that group than in the overweight group (0.4, 95% confidence interval (CI) 0.2, 0.5; *P*<0.001). Among diabetic persons scores were highest in the overweight group, though non-significantly higher than in the normal-weight group (0.5, 95% CI 0.3, 1.2; *P*=0.19). The adjusted difference between non-diabetic and diabetic persons in the difference in PCS-12 scores for the overweight versus the normal-weight category was significant (0.8, 95% CI 0.1, 1.6; *P*=0.03). The overall BMI category × diabetes status two-way interaction term also was significant (F(4445)=6.63; *P*<0.001).

For the MCS-12 (Table 3 and Figure 1, panel b), among non-diabetic persons, mean scores were highest in overweight persons, and significantly higher than in obese persons (0.3, 95% CI 0.1, 0.5; *P*=0.01). In diabetic persons, scores were also highest in the overweight group but non-significantly higher than in the obese group (0.0, 95% CI −0.6, 0.6; *P*=0.94). The adjusted difference between non-diabetic and diabetic persons in the difference in MCS-12 scores for the overweight versus the normal-weight category was not significant (−0.3, 95% CI −0.9, 0.4; *P*=0.43). The overall BMI category × diabetes status interaction term also was not significant (F(4445)=1.68; *P*=0.15).

In analyses exploring moderation of the associations of health status (PCS-12 or MCS-12) with BMI category in diabetes by smoking, cancer or time period, none of the respective three-way interaction terms were significant (data not shown, available from the authors).

## Discussion

In a US national sample, we found evidence of a BMI physical health status paradox in diabetes, mirroring the previously described BMI mortality paradox. Consistent with prior work, physical and mental health status scores were lower for diabetic persons as compared with non-diabetic persons in our sample across all study BMI categories.^24, 28, 29^ However, for physical (but not mental) health status, the optimal BMI category was higher among individuals with diabetes (overweight) than among those without diabetes (normal weight). The findings in our diabetes sample are broadly consistent with those of a prior study of Type 2 diabetes, which found that 26 kg m^−^^2^ was the BMI associated with optimal overall health status, measured with the EQ-5D index.^29^ However, no prior studies have compared physical and mental health status by BMI category in concurrent samples of diabetic and non-diabetic persons.

Our findings should not be construed to mean that normal-weight individuals with diabetes should try to gain weight, or that overweight or obese diabetic persons should avoid losing weight.^50^ Also, the differences in physical health status scores that we observed for overweight versus normal-weight diabetic persons are small and unlikely to be clinically significant at the individual level. Prior work suggests that the minimum clinically important difference in scores for the PCS-12 measure is three points,^51^ whereas among diabetic persons we found an adjusted difference of less than one point between the optimal and next most optimal BMI category.

Nonetheless, the findings may have population level implications. Although the observational nature of our analyses precludes causal inference, the findings suggest that diabetes in the context of normal weight may involve more severe physical morbidity than diabetes associated with overweight, possibly driven by genetic and physiological differences between leaner and heavier diabetic persons.^1, 4, 8, 9, 10, 11, 12^ The MEPS data we employed did not permit us to distinguish whether patients had Type 1 or Type 2 diabetes. However, of the 25 million US adults who have diabetes, 90–95% have Type 2 diabetes, suggesting that most of the individuals in our sample reporting diabetes had Type 2 diabetes. Over 85% of persons with Type 2 DM are overweight or obese.^52^ Thus, despite the relatively small differences that we observed in health status scores for heavier versus thinner individuals with diabetes, our findings may have implications for the net public health impact of overweight and obesity in diabetes, particularly Type 2 diabetes.

The study findings may also bear on the long-standing controversy regarding the existence and implications of a BMI mortality paradox in diabetes.^14, 15, 16^ The controversy was newly driven by a recent study by Tobias *et al.,*^13^ in which normal weight was the BMI category of lowest mortality risk in a cohort of persons with incident diabetes. By contrast, all prior studies in this realm found that the overweight or obese diabetic persons had lower mortality risk than their normal-weight counterparts.^1, 2, 3, 4, 5, 6, 7^ Of note, rather than studying a broadly representative sample of individuals with varying durations of diabetes as did prior investigators, Tobias *et al.* studied a selected sample of nurses and physicians with incident diabetes who were free from cardiovascular disease and cancer at diagnosis. Such an approach excludes people with relatively earlier onset and potentially more severe diabetes, which could account for the differing findings of the study relative to others in this realm. In addition, the study by Tobias *et al.* and some others in this realm lacked a concurrent non-diabetic group,^3, 5, 6, 7^ yet such a comparison group is critical to examining the BMI mortality paradox.^43^ Of the two prior studies in this realm that did compare mortality risk by BMI category in concurrent diabetic and non-diabetic samples, both found evidence supporting a BMI mortality paradox in diabetes.^2, 4^ Given that physical health status has been shown to influence mortality risk,^21, 22^ including in diabetes,^20^ our findings suggest possible pathways that could contribute to a BMI mortality paradox.

Some have suggested the apparent BMI mortality paradox in diabetes is created by detrimental health habits (particularly smoking) and pre-existing conditions (especially cancer) leading to both lower BMI and increased mortality.^13, 53^ Thus, we examined whether the interaction of BMI category and diabetes in influencing physical health status was moderated by smoking or cancer. That we found no evidence of moderation by smoking or cancer does not exclude the possibility that they influenced the findings, as our observational study design precludes causal inference. In an attempt to address this issue, some researchers studying the BMI mortality paradox have excluded smokers and many or all persons with comorbid conditions.^13, 53^ We chose not to do so, as it would result in a much smaller, highly selected group of diabetic persons, limiting generalizability and statistical power.^54^ A robust examination of the potential role of smoking or cancer in explaining the BMI mortality paradox will require prospective studies of broadly representative samples that incorporate repeated weight, health status, health habits and comorbidity measurements over time.

Diabetic persons had worse physical and mental health status than non-diabetic persons across all BMI categories. However, the magnitudes of the differences were smaller for mental health status than for physical health status. Further, although decrements in both physical and mental health status associated with diabetes were progressively smaller from the underweight category through the overweight category, decrements in physical health status began to grow larger again in the obese category, whereas decrements in mental health status began to grow larger again only in severe obesity. These findings suggest the possibility of mental health status protective factors among heavier persons. Some genes conferring increased risk for both obesity and diabetes have variants associated with decreased risk for depression.^12, 55^ The findings further suggest that protective effects on mental health in heavier persons, if present, may be offset in severe obesity by detrimental factors. For example, whereas all degrees of increased BMI are stigmatized, in this era of prevalent overweight and obesity, the stigma of severe obesity is orders of magnitude greater, and may affect mental health.^56^ Research is needed to examine these hypotheses.

A strength of our study was the use of national data collected within the past 15 years from concurrent and broadly representative samples of diabetic and non-diabetic persons. Our study also had some limitations. As noted previously, the study was observational, so causal associations cannot be inferred, and the findings are susceptible to unmeasured confoundings, which could differ by diabetes status. Diabetes status was self-reported, and BMI was derived from self-reported height and weight. Prior studies suggest a complex relationship between self-reported and objectively measured BMI, with differences in BMI category misclassification resulting from self-reports based on socio-demographic characteristics (for example, country of residence, sex, race/ethnicity) and BMI category (for example, tendency to underestimate BMI among higher BMI persons versus overestimate BMI among lower BMI individuals).^57, 58, 59^ Further, people who perceive themselves as being normal weight are less likely to report impaired health status than those who perceive they are overweight, regardless of actual BMI.^60^ The net effects of such relationships on the BMI health status associations we observed are uncertain. Studies employing objectively measured BMI and diabetes are required to explore these issues. To explain the contrasting BMI health status relationships we observed for diabetic versus non-diabetic persons, there would also have to be a differential reporting bias by self-reported diabetes status.

In conclusion, in comparing physical and mental health status by BMI category in concurrent national samples of diabetic and non-diabetic persons, we found evidence of a physical (but not mental) health status paradox in diabetes. Physical health status was most optimal in the overweight category among diabetic persons, versus in the normal-weight category among non-diabetic persons. Given that physical health status influences mortality risk, the findings suggest possible pathways to a BMI mortality paradox in diabetes.

## Figures and Tables

**Table 1:** Characteristics of the study sample

*Characteristic*	*Respondents without diabetes* N=*108 518*	*Respondents with diabetes* N=*10 643*	*All respondents* N=*119 161*
Age, years, mean (SE)	44.7 (0.1)	59.9 (0.2)	45.9 (0.1)
Female, % (SE)	50.9 (0.1)	49.9 (0.6)	50.8 (0.1)
			
*Race/ethnicity, % (SE)*			
Hispanic (any race)	13.0 (0.5)	13.7 (0.7)	13.1 (0.5)
			
*Non-Hispanic*			
White	69.7 (0.6)	64.0 (0.8)	69.2 (0.6)
Black	10.7 (0.4)	15.5 (0.6)	11.1 (0.4)
Other race	6.6 (0.3)	6.7 (0.4)	6.6 (0.3)
			
*Household income as % of FPL, % (SE)*			
<100%	11.3 (0.2)	14.5 (0.5)	11.5 (0.2)
100–124%	4.0 (0.1)	6.1 (0.3)	4.2 (0.1)
125–199	13.3 (0.2)	16.9 (0.5)	13.6 (0.2)
200–399	31.0 (0.2)	30.0 (0.6)	31.0 (0.2)
⩾400	40.3 (0.4)	32.5 (0.7)	39.7 (0.4)
			
*Health insurance, % (SE)*			
Private	71.6 (0.4)	60.5 (0.7)	70.7 (0.4)
Any public	13.5 (0.2)	31.6 (0.6)	15.0 (0.2)
None	14.9 (0.3)	7.9 (0.3)	14.3 (0.2)
			
*Education level, % (SE)*			
<High school	5.2 (0.1)	12.5 (0.4)	5.8 (0.1)
Some high school	11.0 (0.2)	14.3 (0.4)	11.2 (0.2)
High school graduate	31.5 (0.3)	34.1 (0.6)	31.7 (0.3)
Some college	24.2 (0.2)	21.6 (0.5)	24.0 (0.2)
College graduate	28.1 (0.4)	17.6 (0.5)	27.3 (0.4)
			
*US census region, % (SE)*			
Northeast	18.4 (0.6)	17.6 (0.7)	18.4 (0.5)
Midwest	22.6 (0.6)	21.4 (0.7)	22.5 (0.6)
South	36.2 (0.8)	40.2 (0.9)	36.5 (0.8)
West	22.8 (0.8)	20.9 (0.8)	22.6 (0.8)
Urban residence,^a^ % (SE)	83.2 (0.7)	79.5 (1.1)	82.9 (0.7)
Chronic conditions,^b^ mean (SE)	0.68 (0.01)	1.79 (0.02)	0.77 (0.01)
Smoker, % (SE)	21.1 (0.2)	16.6 (0.5)	20.7 (0.2)
			
*BMI (kg m^−^*^*2*^*) category, % (SE)*			
<20, underweight	5.9 (0.1)	1.2 (0.1)	5.6 (0.1)
20–<25, normal	33.3 (0.2)	14.9 (0.4)	31.9 (0.2)
25–<30, overweight	35.5 (0.2)	30.7 (0.5)	35.1 (0.2)
30–<35, obese	16.0 (0.1)	26.6 (0.5)	16.9 (0.1)
⩾35, severely obese	9.2 (0.1)	26.5 (0.5)	10.6 (0.1)
			
*Health status, mean (SE)*			
Physical: PCS-12	50.4 (0.1)	40.2 (0.2)	49.6 (0.1)
Mental: MCS-12	51.0 (0.1)	49.0 (0.1)	50.8 (0.1)

Abbreviations: BMI, body mass index; FPL, Federal Poverty Level; MCS-12, SF-12 Mental Component Summary score; PCS-12, SF-12 Physical Component Summary score; SE, standard error.

a Defined as residing in a Metropolitan Statistical Area.

b From a count of eight conditions: cancer, hypertension, coronary heart disease, myocardial infarction, cerebrovascular disease, asthma, emphysema and arthritis.

**Table 2:** Unadjusted physical and mental health status by body mass index category

*Health status measure*	*BMI (kg m^−^*^*2*^*) category*	*Respondents without diabetes* N=*108 518*	*Respondents with diabetes* N=*10 643*	*All respondents* N=*119 61*
Physical: PCS-12, mean (SE)				
	<20, underweight	50.5 (0.2)	37.9 (1.2)	50.3 (0.2)
	20–<25, normal	51.9 (0.1)	41.4 (0.4)	51.5 (0.1)
	25–<30, overweight	50.7 (0.1)	42.3 (0.3)	50.1 (0.1)
	30–<35, obese	48.9 (0.1)	40.3 (0.3)	47.8 (0.1)
	⩾35, severely obese	46.1 (0.1)	37.2 (0.3)	44.3 (0.1)
Mental: MCS-12, mean (SE)				
	<20, underweight	50.1 (0.1)	46.4 (1.1)	50.1 (0.1)
	20–<25, normal	51.1 (0.1)	49.0 (0.3)	51.1 (0.1)
	25–<30, overweight	51.4 (0.1)	49.9 (0.2)	51.3 (0.1)
	30–<35, obese	50.8 (0.1)	49.5 (0.2)	50.6 (0.1)
	⩾35, severely obese	49.2 (0.1)	47.5 (0.3)	48.9 (0.1)

Abbreviations: BMI, body mass index; MCS-12, SF-12 Mental Component Summary score; PCS-12, SF-12 Physical Component Summary score; SE, standard error.

**Table 3:** Adjusted physical and mental health status by body mass index category^a^

*Health status measure*	*BMI (kg m^−^*^*2*^*) category*	*Respondents without diabetes* N=*108 518*	*Respondents with diabetes* N=*10 643*
Physical: PCS-12, mean (SE)			
	<20, underweight	49.5 (0.1)	44.3 (1.0)
	20–<25, normal	50.6 (0.1)	47.6 (0.3)
	25–<30, overweight	50.3 (0.1)	48.0 (0.2)
	30–<35, obese	49.1 (0.1)	46.1 (0.2)
	⩾35, severely obese	46.9 (0.1)	43.1 (0.2)
Mental: MCS-12, mean (SE)			
	<20, underweight	50.2 (0.2)	47.2 (1.1)
	20–<25, normal	50.9 (0.1)	49.3 (0.3)
	25–<30, overweight	51.2 (0.1)	50.2 (0.2)
	30–<35, obese	51.0 (0.1)	50.2 (0.2)
	⩾35, severely obese	50.0 (0.1)	49.1 (0.2)

Abbreviations: BMI, body mass index; MCS-12, SF-12 Mental Component Summary score; PCS-12, SF-12 Physical Component Summary score; SE, standard error.

a Analyses adjusted for age, age squared, gender, race/ethnicity, family income, education, insurance coverage, number of chronic health conditions, smoking status, rurality, region and panel year.
